# The cortisol axis and psychiatric disorders: an updated review

**DOI:** 10.1007/s43440-025-00782-x

**Published:** 2025-09-16

**Authors:** Mina Y. George, Sherif S. Abdel Mageed, Daniel E. Mansour, Sylvia F. Fawzi

**Affiliations:** 1 Department of Pharmacology and Toxicology, Faculty of Pharmacy, Ain Shams University, Cairo, 11566 Egypt; 2 Department of Pharmacology and Toxicology, Faculty of Pharmacy, Badr University in Cairo (BUC), Cairo, Egypt; 3 Medical School, University of Minnesota, Minneapolis, MN 55455 USA

**Keywords:** Cortisol, Depression, Attention deficit hyperactivity disorder, Autism, Anxiety, Bipolar disorder

## Abstract

Cortisol, a key hormone in the body’s stress response, plays a complex role in the development and progression of various psychiatric illnesses. Elevated or dysregulated cortisol levels are linked to mood disorders, including anxiety and depression, where both high and low cortisol can impair cognitive function and emotional stability. In children, chronic stress and high cortisol exposure can lead to long-term behavioral problems, affecting memory, decision-making, and emotional regulation, especially when early-life adversity is present. Similarly, in adults, prolonged cortisol dysregulation often contributes to cognitive decline, mood instability, and an increased vulnerability to psychiatric conditions like major depressive disorder and bipolar disorder. Structural changes in stress-sensitive brain regions, such as the hippocampus and amygdala, are common, as chronic cortisol exposure can cause atrophy in these areas, affecting memory, fear responses, and executive function. This relationship underscores the critical role of managing stress and cortisol levels to prevent or alleviate psychiatric symptoms across the lifespan. In this review, the authors highlight the latest findings regarding the cortisol-psychiatric illness relationship and the available treatment options involving this target.

**Graphical abstract:**

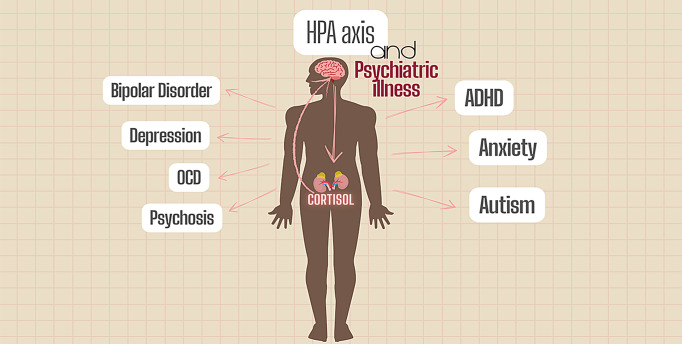

## Introduction

**Fig. 1:**
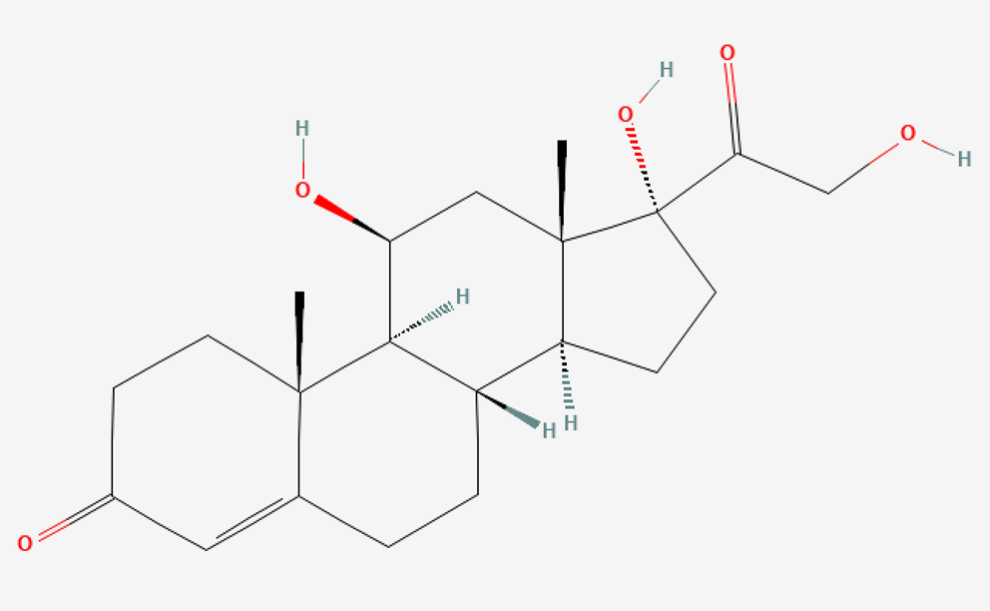
Cortisol structure (8S,10 R,11S,13S)-9,11,12,12-tetradeuterio-11,17-dihydroxy-17-(2-hydroxyacetyl)-10,13-dimethyl-1,2,6,7,8,14,15,16-octahydrocyclopenta[a]phenanthren-3-one

Cortisol (C_21_H_30_O_5_, Fig. 1) is a primary glucocorticoid produced by the adrenal cortex’s zona fasciculata [1]. Its release is controlled by both corticotropin-releasing hormone (CRH) from the hypothalamus and adrenocorticotropic hormone (ACTH) from the pituitary gland through the hypothalamic-pituitary-adrenal (HPA) axis [2]. Cortisol is a stress hormone that regulates the body’s reaction to physical and/or mental stress and helps maintaining the blood pressure, the immune system, the anti-inflammatory response, and the metabolism of protein, glucose, and adipose tissue [3]. It follows a diurnal cycle and increases in response to stress and low blood-glucose concentration [4].

Since the dysregulation of HPA axis activity and elevated cortisol levels according to many studies are strongly associated with the onset and progression of major mental disorders, including depression, bipolar disorder, and psychosis and others, this review article seeks to gather current knowledge regarding cortisol’s complex function in psychiatric pathophysiology, while suggesting specific therapy techniques by evaluating useful pharmaceutical agents, analyzing HPA dysregulation mechanisms, and creating cortisol-guided treatment algorithms by merging clinical trial data with experimental models. This review consolidates and examines the evident impact of cortisol on psychiatric disorders, as well as the research conducted on targeting the cortisol pathway and the HPA axis in the treatment of the associated psychiatric illnesses.

Behavioral problems such as aggression, anxiety, and depression may occur due to chronically elevated levels of cortisol in children. Initially, high cortisol levels may correspond with the onset of these problems, but over time, children with prolonged behavioral issues may exhibit lower cortisol levels due to a blunted stress response [5]. Childhood adversity and trauma are associated with changes in cortisol reactivity, which can negatively impact executive functions and cognitive performance. Children exposed to stress may show diminished cortisol responses to challenges, which is linked to long-term health issues, including obesity and addiction [6]. Chronic stress in childhood can lead to structural changes in the brain, particularly in the hippocampus and prefrontal cortex (PFC), affecting memory and decision-making abilities. These changes can have lasting effects on mental health into adulthood [7]. Serafini et al. [8] concludes that there is a positive association between bullying victimization/perpetration and non-suicidal self-injury (NSSI). This relationship also extends to suicidal behavior. Bullying perpetration was associated with NSSI or suicide attempts only. The relationship was partially mediated by depression and independent of peer rejection. Bully-victims were more involved in NSSI at high levels of peer rejection. The association may be related to impulsiveness, anger, sensation-seeking, or self-punishment. These findings could help detect and recognize individuals at risk for NSSI and suicidal behaviors. In sum, bullying contributes to NSSI largely through its impact on depression, making depression both a critical risk factor and a potential target for interventions aimed at reducing self-injury in individuals affected by bullying.

**Fig. 2:**
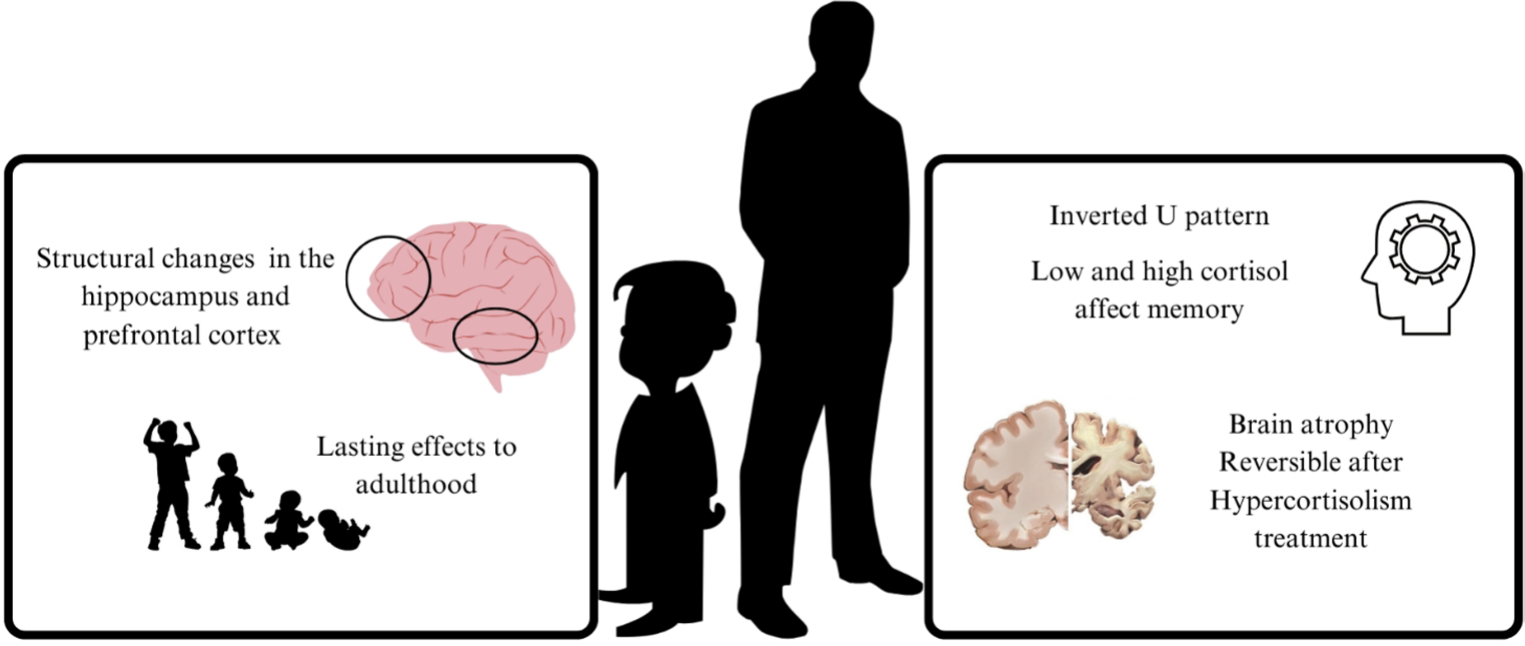
Effect of chronic high cortisol levels on brain structure and function in childhood and adulthood

In adults, particularly those with conditions like Cushing’s syndrome, elevated cortisol is strongly associated with mood disorders such as depression and anxiety. Even slightly elevated cortisol levels can lead to significant mental health issues, including cognitive impairments and increased disability related to mental illness [9]. Chronic high cortisol in adults can lead to cognitive decline, particularly affecting memory and executive function. The relationship between cortisol levels and cognition often follows an “inverted U” pattern, where both low and high cortisol levels can impair cognitive performance, but moderate levels may enhance certain cognitive functions [10]. Thus, Adults may experience a more pronounced impact of chronic stress on the HPA axis, leading to persistent dysregulation of cortisol levels. This dysregulation can contribute to a range of psychiatric symptoms and disorders, including an increased risk for anxiety and depressive disorders. Chronic exposure to excess glucocorticoids can cause cognitive and psychological impairment, including cerebral atrophy in patients with Cushing’s syndrome. Brain volume loss was significantly increased in Cushing patients, but is partially reversible after hypercortisolism correction (Fig. 2). [11]. The factors predicting recovery and mediating the outcomes include duration of Hypercortisolism. Longer duration of exposure to elevated cortisol correlates with more severe brain volume loss and slower or incomplete recovery [12]. Patients with shorter disease duration, e.g., children or adolescents with less than a few years of hypercortisolism, show greater and quicker reversibility of brain atrophy. Another factor is the age of the patient. Younger patients tend to experience faster, and more complete reversal of brain atrophy due to higher neuroplasticity [13]. Adults show partial reversal, often requiring years for improvement with some residual deficits. Furthermore, the extent of brain damage at diagnosis can affect the degree of recovery; severe initial damage may limit complete reversal [12]. Also, brain volume improvements generally begin within one to two years following cortisol normalization and continue over three to four years, with diminishing returns thereafter [14].

Amerio et al. [15] provided detailed insights into the impact of the COVID-19 pandemic on the mental health of vulnerable populations, particularly the elderly and people with chronic long-term health conditions. The World Health Organization declared COVID-19 a Public Health Emergency and later a pandemic, prompting stringent public health measures such as lockdowns, school closures, and quarantine policies to control the spread of the virus. These measures aim not only to curb population-level transmission but also to protect high-risk groups, especially the elderly and those with chronic illnesses, who have a significantly higher risk of severe COVID-19 outcomes and higher mortality rates, e.g., 25% fatality rate in those over 80 years old compared to less than 1% in people under 50. The pandemic has overwhelmed healthcare systems, leading to shortages of hospital beds, medical staff, and equipment, which has negatively affected the care of chronic conditions unrelated to COVID-19. Consequently, standard care protocols for these patients have often had to be modified or reconsidered. Beyond the physical health risks, elderly and chronically ill patients are at increased risk for negative mental health consequences from social distancing and confinement measures. The fear of infection, high fatality risk, and isolation can lead to psychiatric decompensation or worsening of existing mental disorders. Confinement and social distancing can promote feelings of loneliness, hopelessness, despair, and death anxiety, which are known independent predictors of suicide. Access to regular outpatient mental health services has been hindered during the pandemic, creating barriers for patients needing clinical evaluation and medication management. Vulnerable populations may also face exacerbated social stigmatization and marginalization due to pandemic-related social deprivation, uncertainty, and misinformation, potentially leading to segregation and loss of autonomy—factors detrimental to resilience. The authors emphasized the importance of tele-mental health services to maintain care continuity at the community level. Teleconsultations offer a way to support vulnerable individuals remotely, helping them cope with isolation and psychological distress while reducing the burden on hospital-based services and minimizing infection risk. Protecting the mental health of vulnerable groups is a critical public health priority. The authors call for comprehensive, individualized assessments of risk and protective factors to identify specific care needs. They advocate for the design and implementation of targeted mental health interventions tailored to the unique challenges faced by vulnerable populations during the COVID-19 crisis.

The HPA axis is a regulatory system that integrates neuronal and endocrine functions. It comprises the hypothalamus, pituitary, and adrenal cortex tissues, and their associated inputs, releasing factors, and hormones. The paraventricular nucleus of the hypothalamus secretes CRH and arginine vasopressin (AVP), triggering the release of ACTH from the anterior lobe of the pituitary [2]. ACTH stimulates the release of cortisol from the adrenal cortex, which has various central and peripheral effects. It coordinates circadian events, aids in stress coping and recovery, and promotes learning and memory processes. Cortisol is the final product of the HPA axis, and its effects are regulated by two specialized intracellular steroid receptor family subtypes [16]: type I mineralocorticoid receptor (MR) and type II glucocorticoid receptor (GR). Cortisol diffuses through the cellular membrane, binding to receptors, promoting their translocation to the nucleus. Within the nucleus, activated receptors interact with transcription factors and DNA, promoting gene expression [17]. Hypersecretion of CRH may be due to impaired feedback mechanisms from reduced number or function of GRs, as demonstrated in post-mortem studies of patients with severe mood disorders and in mice with acquired forebrain-specific disruption of GR, which mimics major depressive disorder (MDD), and some of these abnormalities are normalized by chronic treatment with imipramine [18]. Furthermore, glucocorticoid sensitivity in mood disorders, particularly MDD and bipolar disorder (BD), was studied. Individuals with mood disorders showed a higher cortisol awakening response/rise (CAR), indicating hyperactivity of the HPA axis. Also, challenge tests using the dexamethasone suppression test (DST) or dexamethasone/CRH revealed mild resistance to glucocorticoids during mood episodes. Higher mean cortisol levels in hair samples were observed in mood disorders [19], providing a retrospective measure of chronic cortisol exposure. Despite elevated cortisol levels, individuals with mood disorders exhibited mild resistance to glucocorticoids. This resistance can be linked to genetic polymorphisms in genes encoding receptors and proteins involved in HPA axis regulation, such as the GR, MR, and CRH receptor-1. Epigenetic changes, along with genetic factors, influence the set-point and regulation of the HPA axis. Early life stress (ELS) [20], such as childhood trauma, and influences during fetal development appear to be important factors in determining an individual’s glucocorticoid sensitivity and risk of developing mood disorders.

Higher levels of cortisol are associated with smaller hippocampal volume, both at baseline and over time. This negative relationship between cortisol and hippocampal volume has been observed in both healthy older adults and patients with mild cognitive impairment or Alzheimer’s disease. Specifically, individuals with higher cortisol levels show faster rates of hippocampal atrophy compared to those with lower cortisol. The faster hippocampal volume decline associated with higher cortisol is linked to poorer memory performance and cognitive deficits over time [21, 22]. However, the relationship between cortisol, hippocampal volume, and cognition is complex [23], as some studies have not found a direct link between cortisol levels and cognitive impairment. The detrimental effects of cortisol on the hippocampus may be due to its neurotoxic properties, leading to neuronal loss and dendritic atrophy. Chronic exposure to high cortisol levels can disrupt the normal functioning of the HPA axis, perpetuating a cycle of stress and cortisol release. High cortisol levels are associated with changes in the amygdala, which is involved in emotional processing and fear responses. Chronic stress and elevated cortisol can lead to functional atrophy of the amygdala, impairing emotional regulation and fear responses. The limbic system, which includes the hippocampus and amygdala, is particularly sensitive to cortisol. High cortisol levels lead to structural and functional changes in these regions [7], contributing to cognitive and emotional impairments. Cortisol partially mediated the effects of genetic variation and life stress on left amygdala volume. Stress-related genetic and early environmental factors contribute to individual differences in stress cortisol reactivity and limbic brain volumes in children, which are phenotypes associated with depression risk in adulthood [24] (Table 1). ELS is associated with increased volume and hyperactivity of the amygdala. The earlier the onset of maltreatment or adversity, the greater the magnitude of amygdala hyperactivity observed. ELS may lead to precocious maturation and increased synaptic plasticity in the amygdala, particularly in males. The structural and functional changes in the amygdala due to ELS are linked to an enhanced vulnerability to developing anxiety, depression, and other psychopathologies later in life [27–29]. In addition to the amygdala, ELS has also been shown to reduce the volume of the hippocampus. The reduced hippocampal volume contributes to the cognitive deficits and emotional dysregulation observed in individuals with a history of early adversity. The effects of ELS on the amygdala and hippocampus may be mediated by stress-induced changes in neurotransmitter systems, synaptic plasticity, and structural connectivity within the corticolimbic circuitry. Genetic and epigenetic factors can also influence the magnitude and persistence of the structural and functional changes in these brain regions following ELS.

**Table 1:** Effect of cortisol on different brain regions [25, 26]

Brain region	Effect of cortisol
Hippocampus	Smaller hippocampal volume, both at baseline and over time
	Faster hippocampal atrophy and poorer memory performance
	Neuronal loss and dendritic atrophy
	Cortisol boosts connectivity within the hippocampus to enhance emotional memory encoding
Dentate gyrus	Neurogenesis suppression and subsequent atrophy
Amygdala	Functional atrophy impairing emotional regulation
	Cortisol mediates effects of genetic variation and life stress on amygdala volume
Frontal lobe	Functional atrophy impairing thought processing and executive function
Orbitofrontal cortex	Reduced grey matter volume in this region with high chronic stress over 20 years

The effect of high cortisol levels on different brain regions, the hippocampus, dentate gyrus, amygdala, frontal lobe, and orbitofrontal cortex

A significant proportion of patients with Cushing’s syndrome experience depression, with prevalence rates reported between 55 and 90%. Depression is often the most prominent psychiatric disorder in these patients. Also, elevated cortisol levels are linked to increased anxiety symptoms. Anxiety disorders have been reported in approximately 12% of patients with Cushing’s syndrome [30]. Chronic high cortisol can lead to cognitive deficits, including problems with memory and concentration. This is particularly relevant in the context of the hippocampus, which is sensitive to cortisol’s neurotoxic effects. Furthermore, patients may also experience mania or hypomania (3–27%), confusion (1%), and psychosis (8%) as part of the psychiatric manifestations of hypercortisolism [30].

Chronic stress and elevated cortisol levels can have detrimental effects on the structure and function of the PFC, impairing its ability to make optimal decisions [31]. Chronic high cortisol leads to dendritic retraction and reduced spine number in the PFC. Acute stress reduces PFC function by disrupting intracellular signaling pathways. It reduces cognitive flexibility facilitated by the PFC, increasing perseverance. It also increases amygdala and striatal control over PFC, facilitating habit-directed learning. Chronic stress is associated with reduced PFC volumes [32]. Elevated cortisol boosts risky decision-making behavior. Men tend to engage in more risk-taking when cortisol levels are high, while in females, in contrast, cortisol does not appear to enhance risk-taking. Some studies suggest that increased cortisol may even diminish risk-taking behavior in females, potentially due to decreased reward responsiveness under stress [33].

Therefore, both acute and chronic stress, mediated by cortisol, can impair PFC structure, reduce its cognitive flexibility, and alter its interactions with other brain regions involved in decision-making. This can lead to riskier, more persevering choices. The effects of stress on PFC decision-making likely involve complex interactions with reward processing and dopamine systems that require further investigation.

## Cortisol and attention deficit hyperactivity disorder

There is a link between cortisol levels and attention deficit hyperactivity disorder (ADHD), though this relationship is intricate and not elusive. Children with ADHD tend to have lower basal salivary cortisol levels in comparison to healthy counterparts. However, no distinct relation between cortisol levels and the severity of ADHD symptoms has been established [34]. Moreover, low hair cortisol concentrations (HCC) have been linked to inattentive ADHD (ADHD-I) symptoms and impaired working memory. Additionally, lower HCC was found at age four to five years to predict the development of ADHD by age eight [35].

A meta-analysis has concluded that children and adolescents with ADHD experience lower morning, random, and cumulative daily cortisol levels in comparison to healthy controls. This suggests that diurnal cortisol may not be a biomarker of ADHD [36]. In contrast, other studies have found no differences in cortisol levels between ADHD and control groups [37, 38]. Children and adolescents with ADHD, especially the combined subtype (ADHD-C) and predominantly hyperactivity-impulsive subtype (ADHD-HI), tend to have lower morning and evening cortisol levels compared to healthy controls. This blunted cortisol response may be related to an underactive behavioral inhibition system, which is closely associated with deficits in self-regulation, working memory, and other core ADHD symptoms [39, 40]. ADHD-I appears to show an elevated cortisol response to psychosocial stress, in contrast to the blunted response seen in ADHD-C [40]. Also, a study reported a trend towards higher cortisol responses in the ADHD-I subtype compared to controls [38]. Long-term cortisol secretion, assessed through HCC, was found to be significantly lower, particularly in boys with ADHD-I. This association persisted even after accounting for the presence of comorbid oppositional defiant disorder, conduct disorder, and anxiety or depressive disorders [35].

In children with ADHD who experience gastrointestinal symptoms, altered cortisol profiles were found. Both hair and salivary cortisol levels were found to be elevated in children with gastrointestinal symptoms [41], indicating a potential link between stress and gastrointestinal issues in these populations. Chronic stress may contribute to or exacerbate gastrointestinal symptoms. Moreover, children with gastrointestinal symptoms showed greater externalizing, e.g., aggression, hyperactivity, and internalizing, e.g., anxiety, depression problems compared to those without gastrointestinal symptoms. The HPA axis-ADHD relationship seems to be complicated and not fully understood. The HPA axis dysfunction may manifest differently across ADHD subtypes. The lower cortisol levels in ADHD, particularly in the ADHD-HI, were found to be more strongly associated with the core symptoms of hyperactivity, impulsivity, and inattention rather than cognitive deficits [39].

Evidence suggests a complex and inconsistent relationship between cortisol regulation and ADHD. This indicates that cortisol alone may not serve as a reliable biomarker as it depends on the disease subtype. Comorbid conditions, such as gastrointestinal issues, appear to further alter cortisol levels and may exacerbate behavioral symptoms. The effects of stimulant medication on cortisol are mixed—some studies show increases, particularly in boys and the combined subtype, while others report no significant changes. Animal studies on prenatal stress support the idea that maternal cortisol exposure may contribute to ADHD-like traits in offspring.

Overall, these findings point to substantial heterogeneity in cortisol patterns across ADHD presentations. Future research should consider ADHD subtypes, sex differences, and comorbidities to better understand the role of HPA axis dysfunction in ADHD.

In their study, VarmiŞ et al. [42] reported that cortisol levels in patients with ADHD measured before and after six months of methylphenidate (MPH) treatment, which inhibits the reuptake of dopamine and norepinephrine, were markedly elevated. In addition, sex differences affect this phenomenon, which was more pronounced in boys with ADHD relative to girls. A significant increase in serum levels of dehydroepiandrosterone (DHEA) and its sulphate ester (DHEA-S) was found after three months of MPH treatment in children with ADHD, but no significant changes in cortisol levels [43]. There is limited evidence that directly targeting cortisol levels can improve ADHD symptoms. It was found that stimulant medications like MPH used to treat ADHD may increase cortisol levels in certain patients, especially boys and those with the ADHD-C subtype. However, the effects are mixed, with other studies finding no significant changes in cortisol with MPH treatment [36].

Children and adolescents with ADHD typically show lower basal morning cortisol, compared to the corresponding controls. The lower activity of the HPA axis and the blocked diurnal cortisol rhythm are linked to physiological under-arousal and may contribute to symptoms such as fatigue and delayed awakening. The stimulant drugs may occasionally normalize cortisol levels, but they tend to revert after long-term treatment [38].

Maternal stress during pregnancy is linked to adverse effects on fetal development and can lead to psychiatric issues in children, including ADHD. Increased cortisol levels in mothers are associated with dysregulation of the HPA axis, which affects both maternal and infant health. Jeon et al. [44] found that administering corticosterone (20 mg/kg/day) to pregnant rats exhibited significantly higher cortisol levels compared to control groups, in both moms and their pups. Moreover, pups displayed hyperactivity and impulsivity, characteristic of ADHD, as evidenced by increased activity in the forced swimming and open field tests, greater exploration in the open arms of the elevated plus maze test, and longer swimming distances in the Morris water maze test, indicating cognitive impairments. In addition, it was found that cortisol-induced pups revealed lower body weight compared to the control ones, suggesting that maternal cortisol exposure adversely affected fetal growth.

Kim et al. [45] treated pregnant rats with corticosterone. Corticosterone treatment affected the offspring’s learning and memory abilities. Furthermore, this memory and learning impairment was linked to a delay in the development of synapses in the CA1 region of the hippocampus. Specifically, corticosterone treatment caused structural and functional alterations in the postsynaptic density and dendritic spines, which are essential for synaptic transmission and plasticity.

In their article, Kozłowska et al. [46] explored the differences in serum steroid hormone levels in the blood of spontaneously hypertensive rats (SHR) as an animal model for ADHD. SHR rats exhibit altered levels of key steroid hormones, including corticosterone, testosterone, and estradiol, compared to control rats. The altered corticosterone levels, indicative of a dysregulated stress response, are particularly significant, as elevated corticosterone is often associated with hyperactivity and attention deficits, common traits in ADHD. These findings suggest that hormonal imbalances in SHR rats may play a role in ADHD-like behaviors, providing insights into the physiological mechanisms that could underlie the condition in humans. The study highlights the importance of hormonal regulation in ADHD pathology and offers a potential avenue for future therapeutic strategies targeting these imbalances.

Combined exposure to deltamethrin, a common pesticide, and corticosterone, during neurodevelopment, resulted in epigenetic changes in male mice. This may be examined by the effects on the *Nr3c1* gene, a gene that encodes the GR in the midbrain region. Male mice exposed to both deltamethrin and corticosterone during critical periods of brain development showed increased hypermethylation of the *Nr3c1* gene. Hypermethylation generally leads to reduced gene expression [47], suggesting that the GR function may be diminished in these mice. The changes in *Nr3c1* methylation are associated with alterations in stress response regulation, which could contribute to the development of neurodevelopmental disorders or affect stress-related behaviors in adulthood.

Together, these studies suggest that cortisol and its regulatory mechanisms—both hormonal and epigenetic—play a significant role in shaping ADHD-related neurodevelopment. While therapeutic interventions targeting cortisol regulation are still in early stages, this line of research may offer promising avenues for novel ADHD treatments, particularly those tailored to stress axis dysfunction. For drugs targeting cortisol in the treatment of ADHD, see Table 2.

**Table 2:** Drugs for treatment of attention deficit hyperactivity disorder by targeting cortisol

Drug	Mechanism of action	Effect on cortisol levels	Ref.
Atomoxetine	Selective norepinephrine reuptake inhibitor	Increases cortisol levels at bedtime compared to unmedicated children	[48]
MPH	Inhibits the reuptake of dopamine and norepinephrine	Increases salivary cortisol levels after one month of treatment.	[49]
Dexmethylphenidate	Isomer of MPH, acts similarly on neurotransmitters	Similar effects on cortisol as MPH	[50, 51]

Atomoxetine, methylphenidate, and dexmethylphenidate for regulating attention deficit hyperactivity disorder symptoms by increasing cortisol levels; MPH: methylphenidate

## Cortisol and autism

Children with autism often show difficulties in adapting to change. A significantly higher serum cortisol response was found in the group of children with autism [52]. Analysis showed significantly higher peak cortisol levels and prolonged duration and recovery of cortisol elevation following the blood-stick stressor in children with autism. Increased reactivity of the HPA axis was noticed in response to stress and novel stimuli in children with autism. Elevated and prolonged cortisol levels have been associated with reduced size of brain regions like the hippocampus in individuals with and without autism spectrum disorder (ASD) [52]. The prolonged cortisol elevation seen in many with ASD appears to be a function of both age and level of social engagement, with older children showing enhanced cortisol responses to relatively benign social situations [53].

Children and individuals with ASD exhibit abnormal cortisol levels and responses, including higher basal cortisol levels, flattened diurnal cortisol rhythms, and exaggerated cortisol responses to stress and novel stimuli [54, 55]. This suggests dysregulation of the HPA axis in ASD. The abnormal cortisol profiles in ASD have been associated with core symptoms like repetitive behaviors, as well as comorbid issues like anxiety and sleep disturbances.

Adolescent transitions may increase risk and vulnerability due to intensified acute physiological arousal, while moderate levels of stress are adaptive and necessary for survival. However, repeated, exaggerated, and prolonged physiological responsivity to stressors can be deleterious and result in pronounced dysregulation of the HPA axis, potentially causing autism [56]. Understanding stress and anxiety in individuals with ASD is crucial for preparing them for this challenging developmental milestone [57].

These findings support the hypothesis that chronic HPA axis dysregulation is both a consequence of and a contributing factor to the behavioral phenotype of ASD. As such, interventions that help regulate stress responses—especially during sensitive developmental transitions like adolescence—may hold therapeutic value. Future research should explore stress modulation strategies tailored to individuals with ASD, particularly those aimed at improving resilience and social adaptability.

Previous reports on cortisol levels in autism are heterogeneous. Some studies revealed altered cortisol rhythms, with either elevated or reduced cortisol responses to stress. In fact, dysregulated HPA axis activity may be linked to anxiety and social impairments common in autistic disorders [58].

There is limited evidence on specific therapies aimed at regulating cortisol levels in individuals with ASD. Very limited data from therapeutic interventions indicated more clinical than biological response. This suggests that while some interventions may impact the clinical symptoms of ASD, they have not been shown to effectively normalize cortisol levels [59]. The focus seems to be more on characterizing the abnormal cortisol profiles observed in ASD, rather than evaluating treatments [52, 60]. One study mentions that fetal cortisol exposure has been identified as a predictor of ASD, but does not discuss therapies to address this [61]. More research is needed to determine the specific HPA axis abnormalities in ASD and whether targeting this system could be an effective treatment strategy (Tables 3 and 4).

**Table 3:** Studies of cortisol and autism relationship

Key findings	Ref.
Lower plasma β-endorphin levels. No significant differences in plasma ACTH or cortisol levels.	[62]
Individuals with autism had significantly lower serum cortisol concentrations. Higher concentrations of ACTH and prolactin.	[63]
A negative relationship was found between morning cortisol levels and parent-reported symptoms of stress on the Stress Survey Schedule. Lower cortisol levels are often seen in conditions of chronic stress and in social situations characterized by unstable social relationships. Sensory processing, auditory filtering, is associated with lower cortisol, while others, visual/spatial sensitivity, were associated with higher cortisol. Increased sensory sensitivity may enhance the susceptibility of children to external factors leading to greater diurnal variability and less regulated cortisol responses.	[64]
Higher peak cortisol levels and prolonged duration and recovery of cortisol elevation. Increased reactivity of the HPA axis to stress and novel stimuli.	[52]
Elevated peripheral cortisol levels.	[61]
There are associations between mothers’ cortisol profiles and children’s sleep problems in ASD.	[65]

Studies that show the correlation found between autism spectrum disorder and cortisol levels, hypothalamic-pituitary-adrenal axis, and any possible findings

ACTH: adrenocorticotropic hormone, HPA: hypothalamic pituitary adrenal, ASD: autism spectrum disorder

**Table 4:** Drugs targeting cortisol in treatment of autism

Drug name	Mechanism of action	Effect on cortisol	Ref.
MPH	Dopamine and norepinephrine reuptake inhibitor	In ADHD with ASD, MPH can ameliorate symptoms related to hyperactivity and inattention in children with ASD by increasing cortisol levels.	[66]
Sodium butyrate	Histone deacetylase inhibitor	Regulates CRHR2 expression and normalizes corticosterone levels; may ameliorate anxiety and social deficits.	[67]

Methylphenidate and sodium butyrate for regulating autism spectrum disorder symptoms by affecting cortisol levels; ADHD: attention deficit hyperactivity disorder, ASD: autism spectrum disorder, MPH: methylphenidate, CRHR-2: corticotropin releasing hormone receptor-2

## Cortisol and anxiety

Anxiety disorders are the most prevalent mental health conditions, typically emerging before or during early adulthood. They are characterized by persistent and impairing symptoms, such as excessive fear, anxiety, or avoidance of perceived threats. These disorders result from dysfunctions in brain circuits responsible for danger response. Genetic factors, hormonal imbalances, environmental influences, and their epigenetic interactions all contribute to the risk of developing anxiety disorders [68]. A positive correlation between cortisol and anxiety has been previously reported in numerous studies encompassing a broad age spectrum. In a study investigating the anxiety and salivary cortisol levels (SCL) in children undergoing esophago-gastro-duodenoscopy (EGD) – a supposedly anxiety-inducing procedure for children [69], SCL before EGD in the patient group was significantly higher than basal and post-EGD values, which was not the case for the control group suggesting a correlation between cortisol levels and anxiety in children. In another study aiming to demystify the effect of HPA axis activity on the development of psychotic disorders in adult high-risk patients [70], it has been proven that salivary cortisol secretion incurred a relationship with affective symptoms; anxiety being one of them. Moreover, the role of cortisol in panic disorder -an anxiety-related disorder- has been further emphasized in a study that showed potential alteration of cortisol responsiveness to stress in panic disorder sufferers. The sufferers and a control group underwent different psychometric and physiological measures while assessing state anxiety and salivary cortisol. The state anxiety test showed a reduction over the course of the experiment for both groups with cortisol levels showing a corresponding decline only in the control group, suggesting a relationship of cortisol with panic disorders and the associated HPA axis dysregulation [71]. This pattern can be explained by an uncoupling of the HPA axis and the Noradrenergic system wherein cortisol levels behave independently of the state of anxiety [72].

Not only has a positive correlation between cortisol and anxiety been reported, but more importantly a unidirectional causal relationship has also been demonstrated. A Mendelian Randomization meta-analysis using variants in the *SERPINA6*/*SERPINA1* -a locus that encodes proteins impacting morning plasma cortisol levels- revealed a causative relationship between cortisol and anxiety [73]. While the exact mechanisms underpinning the role of cortisol in the etiology of anxiety are yet to be elucidated before the clinicopathologic and pharmaceutical significance of cortisol can be defined, this finding makes cortisol an attractive target for the treatment of anxiety-related disorders.

Conversely, some studies reported anxiety and cortisol levels that were independent of each other. In a study aiming to compare the salivary cortisol and anxiety levels in melanoma patients undergoing sentinel lymph node excision under local anesthesia versus general anesthesia [74], patients who underwent local anesthesia exhibited significantly higher cortisol concentrations at the beginning of surgery as well as 20 minutes after incision compared to the general anesthesia group. The anxiety levels were operationalized in this study using the Hospital Anxiety and Depression Scale which the participants completed during various pre- and postoperative periods. Anxiety levels did not significantly differ during pre- and postoperative periods between patients in both groups.

The molecular pathways underlying the causative relationship between cortisol and anxiety remain unknown. However, there have been molecular explanations. Neuroinflammation is an established mechanism underpinning the etiology of anxiety disorders [75]. While cortisol, as a potent anti-inflammatory agent, inhibits the neuroinflammation-mediated development of anxiety disorders, inflammation-induced glucocorticoid resistance can stunt this desirable impact of cortisol, further exacerbating inflammatory processes [76]. Further cortisol secretion might represent the body’s normal response to the lack of responsiveness of inflammation to cortisol which might explain the high levels of anxiety that are paralleled with similar levels of cortisol.

Overall, while there is substantial support for cortisol’s role in the etiology and maintenance of anxiety disorders, the relationship is not always linear or uniform. Variability may stem from individual differences in HPA axis sensitivity, inflammation status, genetic predispositions, and environmental contexts. Future research should prioritize identifying moderators of this relationship and exploring whether cortisol-targeted interventions could yield meaningful therapeutic benefits in anxiety treatment.

Anxiety disorders are always linked to elevated physiological basal cortisol levels and exaggerated cortisol responses to stress, indicating HPA axis hyperactivity. However, some anxiety cases exhibit blocked cortisol response, highlighting variability [77].

There have been pharmaceutical treatments that proved effective at mitigating the cognitive burden associated with anxiety-related disorders. A study by Lenze et al. [78] reported that serotonin-reuptake-inhibitor-induced changes in cortisol levels during acute treatment were found to predict neuropsychological function improvement in older adults with anxiety disorders. More specifically, reductions in cortisol levels due to selective serotonin reuptake inhibitors (SSRIs) were related to changes in immediate and delayed memory in patients receiving treatment compared to those receiving a placebo, suggesting a cortisol-related pharmaceutical implication in mitigating anxiety disorders neurocognitive sequelae

Interestingly, the dynamic between cortisol levels and anxiety extends to include non-human subjects as well. In a study conducted to examine the influence of cortisol levels on anxiety-related behaviors in cattle, cortisol levels were correlated with time spent ruminating and later entrance to the squeeze chute in experimental cattle relative to their non-stressed counterparts [79] (Table 5).

**Table 5:** Drugs targeting cortisol in treatment of anxiety

Drug class	Drug name	Mechanism of action	Effect on cortisol	Ref.
SSRIs	Escitalopram	Inhibits the reuptake of serotonin, enhancing serotonergic neurotransmission. Inhibits CRF release in the amygdala and increases GR density in the hippocampus.	Reduces peak and total cortisol levels in patients with elevated baseline cortisol, particularly in older adults with Generalized Anxiety Disorder	[80]
Benzodiazepines	Clonazepam, alprazolam	Enhance the effect of the neurotransmitter GABA at the GABA-A receptor, leading to increased inhibitory neurotransmission.	Reduces activity of CRF neurons in the hypothalamus, thereby decreasing cortisol secretion	[81]
Tricyclic antidepressants	Amitriptyline, nortriptyline	Inhibit the reuptake of norepinephrine and serotonin, increasing their availability in the synaptic cleft.	Modulates HPA axis activity, potentially lowering cortisol levels during treatment	
Corticosteroids	Hydrocortisone	Mimics cortisol effects; can be used to assess HPA axis function.	Can increase cortisol levels; however, in controlled doses, may help stabilize mood and anxiety symptoms by modulating HPA axis feedback mechanisms.	

Selective serotonin reuptake inhibitors, benzodiazepines, tricyclic antidepressants, and corticosteroids for regulating anxiety symptoms by targeting cortisol levels; SSRIs: selective serotonin reuptake inhibitors, CRF: corticotropin releasing factor, GR: glucocorticoid receptor, GABA: gamma amino butyric acid, HPA: hypothalamic pituitary adrenal

## Cortisol and bipolar disorder

Bipolar disorder (BD) is a severe mood disorder characterized by alternating episodes of mania and depression. The pathophysiology of BD is complex and multifactorial, involving genetic, biochemical, and environmental factors [82]. Cortisol has been implicated in the etiology and progression of BD [83]. Numerous studies have demonstrated that individuals with BD exhibit dysregulation of the HPA axis, leading to abnormal cortisol secretion patterns, which may contribute to mood instability [84]. Elevated cortisol levels, disrupted diurnal rhythms, and altered HPA axis function are commonly observed in BD patients, particularly during mood episodes. Neurobiological mechanisms involving neuroinflammation, GR sensitivity, and neurotransmitter interactions further elucidate the impact of cortisol on mood regulation [84].

Bipolar disorder features variations in HPA axis dysregulation, with many patients showing elevated physiological cortisol levels during manic and depressive patterns. Persistent hypercortisolemia can lead to hippocampal atrophy and cognitive deficits associated with bipolar disorder. Cortisol abnormalities could indicate the severity of mood symptoms and may reflect chronic stress and neurobiological vulnerability [85].

While studies emphasize the significance of cortisol as a biomarker and therapeutic target, it stops short of discussing the variability in findings across patient populations, stages of illness, and treatment response. There is also a lack of critical engagement with potential confounding factors—such as stress, medication effects, and comorbid conditions—that might influence cortisol levels independently of BD. Future research should focus on integrative approaches that address these multifaceted influences to develop targeted treatments for BD (Tables 6, 7).

**Table 6:** Studies of cortisol and bipolar disorder relationship

Study	Participants	Findings	Ref
Circadian secretion of cortisol in BD	18 patients with BD and 5 control subjects	Increased cortisol levels in depressive and manic phases; no significant differences among patient groups Cortisol levels are not state markers in BD.	[86]
Ovarian hormone fluctuation, neurosteroids, and HPA axis dysregulation in perimenopausal depression	Women experiencing perimenopausal depression	Fluctuations in ovarian hormones and their derived neurosteroids lead to alterations in GABAergic regulation of the HPA axis, increasing sensitivity to stress and vulnerability to depression during the menopause transition	[87]
Cortisol levels in unmedicated patients with unipolar and BD	Unmedicated patients with unipolar and bipolar major depression, healthy controls	Lower total daily salivary cortisol output associated with depressive symptoms; no significant difference between unipolar and bipolar depression Hypocortisolemia may be linked to bipolar depression, while hypercortisolemia is more associated with unipolar depression.	[88]
The HPA axis dysregulation in severe mental illness	Patients with BD	Microbial imbalances influence cortisol regulation and mood stability. Gut bacteria produce neuroactive compounds affecting brain function and that stress-induced gut dysbiosis can exacerbate HPA axis dysregulation. Modulating gut microbiota through dietary interventions or probiotics could restore HPA axis balance and improve mood stability in BD patients.	[89]
Effects of childhood trauma, daily stress, and emotions on daily cortisol levels in individuals vulnerable to suicide	142 individuals categorized into three groups based on suicidal history: suicide attempt, suicidal ideation, and control group	Participants in the suicide attempt and ideation groups exhibited significantly lower CAR and tended to have flatter cortisol slopes throughout the day compared to controls. Childhood trauma was associated with lower CAR and influenced suicide vulnerability group membership through its effect on daily CAR levels.	[90]
Pathophysiological implications of neuroinflammation mediated HPA axis dysregulation	Cancer patients experiencing depressive symptoms	Neuroinflammation as a mediator of HPA axis dysregulation in both cancer and depression, suggesting a shared pathway that might also apply to BD.	[91]
Associations between the cortisol awakening response and patient evaluations	Patients with BD	Increased CAR associated with smartphone-based mood instability; no significant correlation with clinician-evaluated symptoms. CAR may reflect daily stress and mood instability in bipolar patients.	[92]
Meditation interventions efficiently reduce cortisol levels of at-risk samples	Various at-risk populations, including patients with somatic illnesses and individuals in stressful life situations – a meta-analysis	Meditation interventions significantly reduced cortisol levels in at-risk samples, with a medium effect size for blood samples and a small effect for saliva samples. Meditation is particularly beneficial for those at risk of elevated cortisol levels.	[93]
The influence of comorbid depression and overweight status on peripheral inflammation and cortisol levels	216 participants: 69 overweight patients with depression, 35 overweight controls, 55 normal-weight patients with depression, 57 normal-weight controls	Overweight patients with depression exhibited significantly higher levels of inflammation [high sensitivity C-reactive protein]. Comorbid depression and overweight status were associated with an increased risk of clinically elevated high sensitivity C-reactive protein levels.	[94]
Chronic Stress in BD	BD patients across various states	HPA axis activity is complex, showing both hyperactivity and hypoactivity related to stress responses.	[95]
Longitudinal hair cortisol in BD	Individuals diagnosed with BD	Persistent cortisol fluctuations trigger mood episodes; emotional reactivity enhances daily stress inputs to the HPA axis, leading to elevated cortisol levels over months.	[96]
HCC in pregnant women with BD	69 women [32 with severe mental disorders, 37 controls)	Elevated HCC in women with severe mental illness compared to controls; poorer symptomatic functioning linked to higher HCC.	[97]

Previous studies that show differences of cortisol levels in relation to bipolar disorder, the mood fluctuations, and the cortisol findings

BD: bipolar disorder, HPA: hypothalamic pituitary adrenal, GABA: gamma aminobutyric acid, CAR: cortisol awakening response/rise, HCC: hair cortisol concentrations

**Table 7:** Drugs targeting cortisol in treatment of bipolar disorder

Drug name	Mechanism of action	Indication	Effect on cortisol	Ref.
Mifepristone	GR antagonist	Psychotic depression, BD	Reduces cortisol levels; shows promise in normalizing HPA axis dysfunction in BD.	[98]
DHEA	Hormone precursor with potential neuroprotective effects	BD, mood stabilization	May help normalize HPA axis activity and cortisol levels; potential antidepressant effects noted.	
Antidepressants	SSRIs, SNRIs	Depression, anxiety in BD	Often indirectly modulate HPA axis activity; may help normalize cortisol levels over time through serotonergic pathways.	[99]
Vasopressin receptor antagonists	Modulates water retention and blood pressure	Affective disorders, including BD	Targeting vasopressin receptors may influence HPA axis activity and cortisol response; potential for mood stabilization.	[100]
Metyrapone	Cortisol synthesis inhibitor	Depression, adjunctive treatment for BD	Reduces cortisol production; may help restore normal HPA axis function and improve mood symptoms.	

Effects of mifepristone, DHEA, antidepressants, vasopressin receptor antagonists, and metyrapone on cortisol regulation in bipolar disorder

GR: glucocorticoid receptor, BD: bipolar disorder, HPA: hypothalamic pituitary adrenal, DHEA: dehydroepiandrosterone, SSRIs: selective serotonin reuptake inhibitors, SNRIs: serotonin norepinephrine reuptake inhibitors

## Cortisol and depression

Depression is one of the most common mental diseases in the world that is a complex and multifactorial disease, most frequently manifested by a depressed mood and reduced psychomotor drive [9]. It is accompanied by circadian rhythm disorders, e.g., sleep disorders, as well as anxiety, pain, weight fluctuations, and other somatic symptoms. Patients often have suicidal thoughts, feelings of guilt, and loss of interest. Depression may also accompany other conditions, such as cardiovascular disease, cancer, hormonal disorders, viral infections, and vitamin deficiencies [9]. The significant influence of cortisol on the body’s metabolism, gene expression, and the central nervous system causes a dysregulation of the HPA axis, and consequently fluctuations in cortisol levels, which strongly affects the mental state of patients. Therefore, cortisol is considered one of the most significant biomarkers of anxiety disorders and depression [101]. It, as the main adrenocortical stress hormone, is known to be hyper-secreted in many depressed individuals. The facts that people with Cushing disease frequently experience depression and anxiety, and also that stress increases glucocorticoid synthesis and release, contributed to the current stress hypothesis of depression [102]. Excess cortisol and other HPA axis hormones, according to this concept, play a crucial role in the etiology of depression [103].

A study showed that cortisol secretion increases regardless of gender, with women experiencing a higher correlation with anxiety disorders than depression. Men with both diagnoses also experienced increased cortisol secretion, corresponding to the severity of symptoms [104]. During remission of the disorder, the activity of the HPA axis was normalized. However, despite the remission and normalization of cortisol secretion, cognitive dysfunction remained in patients with depression or a depressive episode in the past. It may be associated not only with changes in the brain that occur during the disorder but also with the age of the patients [9]. In individuals with MDD, there is often a dysregulation of the HPA axis, resulting in elevated cortisol levels and impaired negative feedback mechanisms that typically help regulate cortisol production [105, 106]. The impaired feedback inhibition of the HPA axis, particularly the dysfunction of GRs, contributes to the persistence of elevated cortisol levels in depressed individuals [106]. ELS, such as trauma or adverse childhood experiences, can program the HPA axis, increasing the risk of developing depression later in life. This programming may result in long-term changes in stress response and cortisol regulation, making individuals more vulnerable to depressive episodes when faced with stress [107].

Elevated cortisol can lead to a deficiency in serotonin, a neurotransmitter that plays a critical role in mood regulation. Chronic high cortisol levels can disrupt the production and function of serotonin, leading to symptoms commonly associated with depression, such as anxiety, insomnia, and apathy [108]. Cortisol has been shown to enhance the expression of the gene coding for the serotonin transporter, leading to increased uptake of serotonin in peripheral blood lymphocytes [109]. This effect suggests that elevated cortisol levels can influence serotonin dynamics, potentially altering its availability in the brain. In studies involving patients with chronic stress and depression, a significant correlation was observed between cortisol levels and serotonin uptake, indicating that high cortisol may modulate serotonin transport mechanisms [110].

Cortisol can also lower plasma levels of tryptophan, the precursor to serotonin. This reduction in tryptophan availability may subsequently lead to decreased serotonin synthesis [111]. Elevated cortisol levels, particularly following stressful life events, have been linked to lower plasma tryptophan levels in individuals, which can contribute to diminished serotonin production and function [112]. It may also interact with serotonin receptors. Elevated cortisol can increase the sensitivity of certain serotonin receptors, such as the 5-HT_2C_ receptor, which could alter serotonergic signaling pathways. This modulation can have complex effects on mood and emotional regulation, potentially contributing to depressive symptoms [113].

In individuals experiencing chronic stress or depression, the capacity for cortisol to further increase serotonin uptake may become limited. Research indicates that while cortisol can initially enhance serotonin transporter activity, this effect may plateau or diminish in hypercortisolemic patients, suggesting a form of desensitization or dysregulation in serotonin transport mechanisms under prolonged stress conditions [114].

Cortisol is known to have neurotoxic effects, particularly on the hippocampus, a brain region crucial for memory and emotional regulation. Prolonged exposure to high cortisol levels can result in hippocampal atrophy, which is associated with cognitive deficits and emotional disturbances seen in depression [115, 116]. This damage may impair the brain’s ability to process emotions and stress, increasing vulnerability to depressive episodes.

In contrast to the anti-inflammatory properties of glucocorticoids through NF-κB suppression, rats subjected to chronic unpredictable stress exhibited heightened glucocorticoid levels, leading to enhanced NF-κB activation and pro-inflammatory gene expression [115]. Cortisol can suppress the production of brain-derived neurotrophic factor (BDNF), a protein essential for neuron growth and survival. Reduced BDNF levels are linked to neurodegenerative processes and have been implicated in the development of depression. Chronic stress and elevated cortisol levels can thus contribute to decreased BDNF, further exacerbating depressive symptoms [117].

The CRF was chemically identified and considerably improved the study of the HPA axis, stress, and depression. Neurons from the hypothalamic paraventricular nuclei project to the median eminence, where their nerve terminals secrete CRF into the hypothalamo-hypophyseal portal system. CRF is then carried to the anterior pituitary via this specialized vascular route, where it works on corticotrophs to stimulate ACTH secretion, hence modulating HPA axis activity. CRF is broadly distributed in extrahypothalamic brain areas, where it works in conjunction with the hypothalamic CRF system as a neurotransmitter coordinating the behavioral, autonomic, endocrine, and immunological responses to stress [118]. Many of the HPA axis changes seen in depressed patients may be caused by persistent CRF hypersecretion. CSF-CRF concentrations in depressed people are often increased.

Other genes involved in the regulation of the CRF and the HPA axis have been related to depression vulnerability associated with ELS. Several single-nucleotide polymorphisms (SNPs) in the CRF1 receptor gene, *CRHR-1*, and their role in regulating adult depression in persons who had ELS in the form of child abuse were also studied. The CRHR-1 receptor is important in the regulation of the HPA axis in response to stress. It mediates the effect of CRF on the pituitary gland, causing it to release ACTH, which promotes cortisol production [119]. Furthermore, CRF activity at the CRHR-1 in extrahypothalamic areas causes anxiety and depression symptoms. Several SNPs and the two haplotypes created by these SNPs, TAT and TCA, affected the association between early adverse experiences and risk of developing depression [119]. The TAT and TCA haplotypes reduced the severity of adult depression symptoms in people who had experienced moderate to severe childhood abuse. The genotypes and haplotypes of CRHR-1 variations may serve as potential predictors of adult depression risk and resilience in men and women with a history of child abuse.

**Fig. 3:**
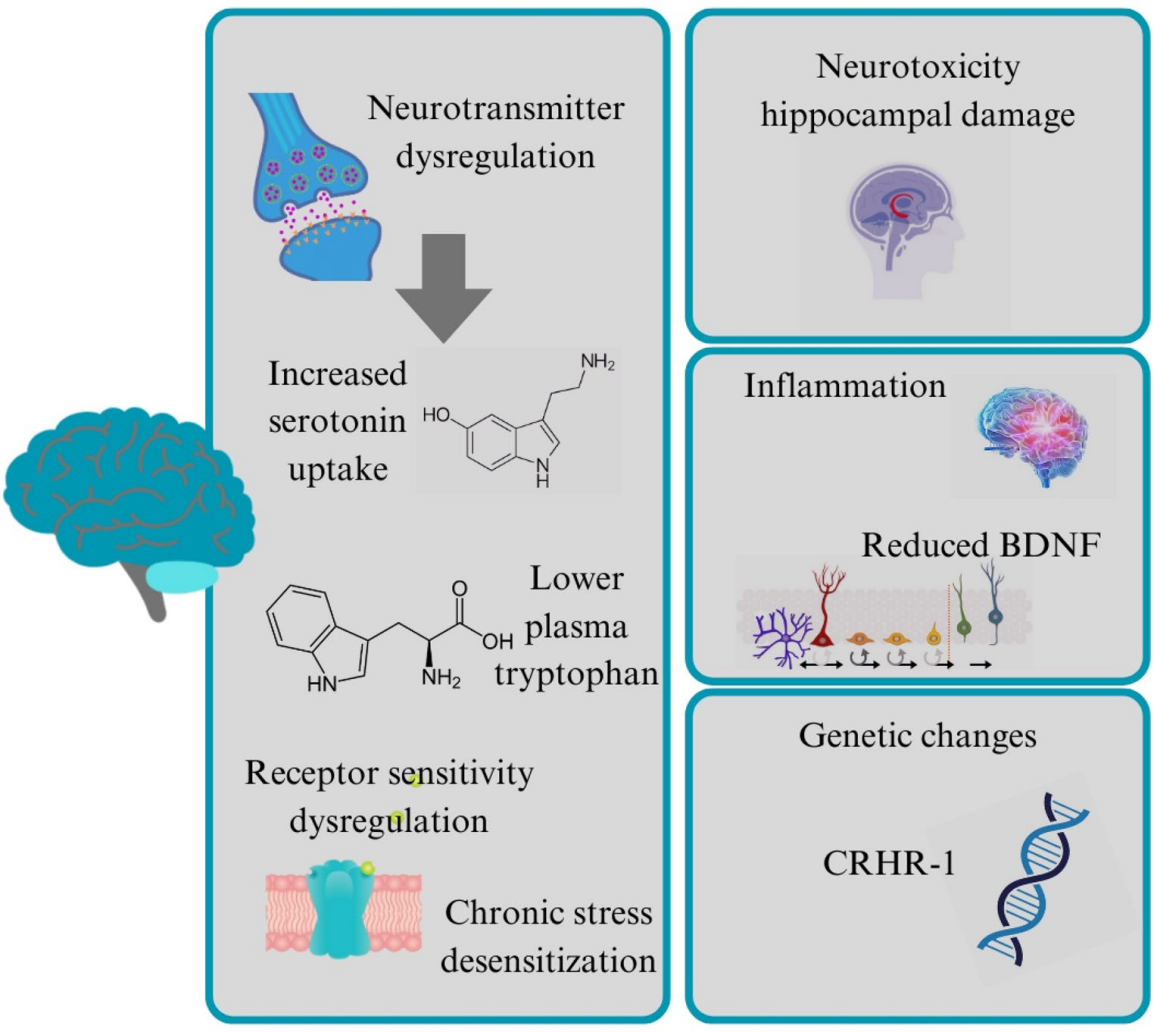
Possible mechanisms underlying depression induced by high cortisol levels; BDNF: brain-derived neurotrophic factor, CRHR-1: corticotropin-releasing hormone receptor 1 gene

The most primarily reported HPA axis changes related with depression included suppressed cortisol circadian rhythms with higher levels late in the day [120], negative feedback insensitivity, as characterized by inability of morning cortisol suppression after dexamethasone administration [121], increased CRF concentrations in the CSF [122, 123], and brain CRHR-1 receptors and mRNA down-regulation. This reflected compensatory changes in response to persistently high CRF concentrations [124]. For all the possible mechanisms underlying cortisol and depression, see Fig. 3.

In conclusion, persistent cortisol elevation, driven by impaired negative feedback and glucocorticoid receptor dysfunction, contributes to both the emotional and cognitive disturbances observed in depression. Moreover, early life stress and genetic variations, particularly in the CRHR-1 gene, appear to shape individual vulnerability by altering stress responsivity and HPA axis regulation. These findings not only reinforce cortisol’s value as a potential biomarker but also open avenues for more targeted and personalized therapeutic strategies aimed at restoring neuroendocrine balance in depressive disorders. Future research should track cortisol levels and HPA axis activity across the different phases of depression, acute, remission, and relapse, to better understand whether these markers fluctuate with symptom severity or represent stable traits. Also, more studies are needed to integrate CRHR-1 polymorphisms and early life stress into predictive models of depression.

Elevated cortisol and activated HPA axis are well-known neurobiological patterns in major depressive disorder. Chronic stress and elevated cortisol lead to hippocampal atrophy, prefrontal cortex alterations, and cognitive impairment associated with depression. Dysregulation of cortisol secretion, including flattened diurnal and increased cortisol awakening response, is common in depression. These changes are parallel to those seen in Cushing’s syndrome, though typically less severe [120].

To target cortisol for treatment of depression, Mifepristone inhibits progesterone, as well as GRs at higher doses. It is a specific GR antagonist that partially or totally inhibits GR agonist binding. Following the mifepristone injection [125], GR levels increase rapidly within hours, possibly restoring normal feedback and resetting the HPA axis. Just a short duration of antagonist treatment may be sufficient to restore normal HPA axis function. Mifepristone has been studied as an additive therapy, as well as a monotherapy in the treatment of MDD, primarily the psychotic subtype [126].

Dexamethasone, a synthetic glucocorticoid, inhibits stress-induced HPA activation largely at the anterior pituitary level. According to one study, tiny quantities of dexamethasone can induce a hypocorticoid condition in the brain, with a relatively enhanced glucocorticoid activity in the periphery [127]. Under the correct conditions, partial exclusion of dexamethasone from the brain along with inhibition of peripheral pituitary-adrenal activity can result in a central hypocorticoid state [126]. A low dose of dexamethasone was found to restore anhedonia, normalize adrenal gland weight and body weight, corticosterone and ACTH levels, and reduce memory impairment in experimental animals, indicating that low doses of dexamethasone for short periods of time may be helpful in alleviating depressive-like features and memory impairment [128]. In a clinical study for the treatment of depression [129], a short course of oral dexamethasone is effective and safe at levels of three to four mg per day for four days and produces antidepressant effects. This action is felt at the pituitary gland level because dexamethasone does not penetrate the CNS or central GR at this dose [125, 127].

Ketoconazole (KTZ) is a common antifungal agent. KTZ serves to treat hypercortisolemia by inhibiting different enzymes involved in adrenal cortisol production. It was found to be contributing to decreases in depression ratings in hypercortisolemic patients but not in non-hypercortisolemic individuals when compared to placebo. The drug was found to be well tolerated and didn’t lead to any notable side effects or laboratory abnormality [130]. Clotrimazole is noted for its involvement in the modulation of the HPA axis and its anti-inflammatory effects, which are followed by the activation of neurogenesis. Its action leads to a decrease in serum cortisol levels and inflammasome biomarkers, alongside an increase in neurogenesis markers. This indicates that clotrimazole not only affects stress hormone levels but also promotes brain health by enhancing neurogenesis, suggesting its potential as a novel antidepressant candidate [131].

Metyrapone plus fluoxetine were found to significantly enhance the levels of extracellular DA metabolites 3,4-dihydroxyphenylacetic acid, homovanillic acid, and a serotonin metabolite, 5-hydroxyindoleacetic acid, compared to fluoxetine alone [132]. Among different mechanisms, elevated levels of extracellular dopamine and serotonin metabolites may play a role in metyrapone’s augmentation of fluoxetine efficacy, which may be critical in the treatment of drug-resistant depression. In a study comparing the effects of adding metyrapone to normal SSRI regimens, it was found that this combination of treatments produced a more fast, effective, and long-lasting treatment response in MDD than SSRI alone [133].

Clinical studies have indicated that R121919 may improve depressive symptoms by reducing cortisol levels and altering stress. R121919 works by blocking the CRH receptors, which play a crucial role in the HPA axis. By inhibiting these receptors, R121919 can potentially reduce the secretion of ACTH and subsequently lower cortisol levels, which are often elevated in individuals with depression [134].

Arginine vasopressin (AVP) is a neuropeptide involved in regulating the HPA axis, especially during stress responses. It has been implicated in the pathophysiology of depression, as elevated levels of AVP are often found in depressed patients. The V_1B_ receptor subtype of vasopressin is particularly important in mediating the effects of AVP on the HPA axis. By antagonizing these receptors, V_1B_ receptor antagonists can potentially reduce cortisol secretion and normalize HPA axis activity [135]. Clinical trials have focused on two specific V_1B_ receptor antagonists, TS-121 and ABT-436. TS-121 has demonstrated a tendency to reduce depression scores in patients with MDD, particularly in those with elevated basal cortisol levels [136]. The efficacy of TS-121 appears to be more pronounced in patients with HPA axis hyperactivity, supporting the hypothesis that V_1B_ receptor antagonists may be especially beneficial for this subgroup [136]. ABT-436 has shown potential in reducing depressive symptoms [135], although the results have been less conclusive compared to TS-121.

Targeting cortisol and the HPA axis for the treatment of depression presents a promising yet complex therapeutic strategy. Current interventions, such as GR antagonists, e.g., mifepristone, glucocorticoids, e.g., dexamethasone, and steroidogenesis inhibitors, e.g., ketoconazole, demonstrate potential in modulating cortisol levels and alleviating depressive symptoms, especially in subgroups with HPA axis dysregulation. However, these approaches also raise questions regarding long-term efficacy, optimal dosing, and the potential for systemic side effects, particularly with agents like dexamethasone and mifepristone that may affect both central and peripheral systems. Additionally, more studies are needed to assess the neuroprotective outcomes of such therapies, especially concerning cognitive recovery and neuroplasticity.

## Cortisol and obsessive-compulsive disorder

Obsessive-compulsive disorder (OCD) is a chronic, disabling psychiatric disorder marked by the occurrence of obsessions or compulsions, affecting nearly 1–3% of the global population [137]. The clinical presentation of OCD is notably diverse, with patients fitting into certain symptom dimensions, however not all patients suffer these disease patterns [138]. OCD seems to be linked to altered cortisol levels, though the exact relationship remains complex and not entirely clear. Several studies have reported that patients with OCD display significantly augmented basal cortisol levels relative to healthy controls.

In a systematic review and meta-analysis to explore the association between cortisol levels and OCD, Sousa-Lima et al. [139] reported that cortisol dysregulation may contribute to the pathophysiology of OCD. The meta-analysis included multiple studies assessing cortisol levels in individuals diagnosed with OCD compared to healthy controls. Results indicated that individuals with OCD generally exhibited elevated cortisol levels, suggesting a hyperactive HPA axis [140]. The findings also showed a correlation between cortisol levels and the severity of OCD symptoms. Higher cortisol levels were associated with more severe obsessive-compulsive symptoms [139], indicating that stress response may play a significant role in exacerbating OCD. The link between elevated cortisol and OCD is ‘bidirectional’ in nature. In other words, elevated cortisol may contribute to the development or worsening of OCD symptoms, while OCD-related stress could further elevate cortisol levels [141]. However, other research has found that sometimes normal cortisol levels were observed in patients with OCD, but these studies are a small number of studies. This may be partly explained by the fact that differences in factors affecting cortisol levels, like methods of measurement, assessment timing, and the presence of comorbid conditions such as depression. It is worth to mention that increased nocturnal secretion of ACTH and cortisol was found in OCD patients [141]. Hyperactivity of the HPA axis, frequently observed in adults with OCD, has also been detected during earlier developmental stages, with a particular emphasis on the early-morning cortisol peak [142]. In contrast, no significant differences in cortisol levels were found between OCD patients and controls, suggesting that methodological differences may influence results [143]. Additionally, it was noted that higher trait anxiety in OCD patients correlated with an increased CAR and altered cortisol suppression following dexamethasone administration [144]. Lastly, some research suggests that OCD patients may experience chronic low cortisol secretion, which could indicate a down-regulation of the HPA axis as an adaptive response to ongoing stress [145].

Concerning the relationship between the CAR as a marker of HPA axis activity and obsessive-compulsive symptoms in healthy individuals, Melia et al. [146] found sex differences in the association between CAR and obsessive-compulsive symptoms. In women, a higher CAR was linked to more pronounced obsessive-compulsive symptoms, including intrusive thoughts and repetitive behaviors. Even in healthy individuals, those without a clinical diagnosis of OCD, higher CAR was associated with mild obsessive-compulsive traits, particularly in women. This suggests that variations in HPA axis activity could be related to subclinical levels of obsessive-compulsive tendencies. The findings align with prior research suggesting that dysregulation of the HPA axis and stress hormones like cortisol play a role in OCD.

Levels of cortisol, DHEA, and DHEA sulfate (DHEA-S) change in patients with OCD [147]. DHEA and DHEA-S are adrenal hormones involved in modulating stress and immune responses. Some patients showed elevated cortisol levels during therapy, indicating a heightened stress response, while others demonstrated more balanced cortisol responses. Lower cortisol reactivity and higher DHEA/DHEA-S levels during therapy were associated with better treatment outcomes [147]. Patients with more stable or lower stress hormone responses during exposure therapy showed greater reductions in OCD symptoms post-treatment. This suggests that a more adaptive stress response may enhance the effectiveness of exposure therapy in OCD.

The bidirectional nature of the cortisol-OCD relationship—where elevated cortisol may worsen OCD symptoms, and OCD-related stress may increase cortisol levels—complicates efforts to draw definitive conclusions. Future research should prioritize consistent methodologies for cortisol assessment, consider individual biological variability such as sex and anxiety traits, and explore the prognostic value of cortisol, DHEA, and CAR in treatment planning.

Studies on cortisol in OCD patients reveal contradictory results, but there is evidence that patients with OCD may exhibit elevated cortisol levels and dysregulated HPA axis activity, especially under stress conditions. Cortisol alterations correlate with anxiety and stress responses in OCD, reflecting hyperresponsivity to stress. Neurobiological changes such as amygdala and frontal cortex abnormalities linked to HPA axis disruption have been reported, suggesting cortisol variation plays a role in OCD pathophysiology [148].

There are no drugs found for the treatment of OCD yet that work on the HPA axis and cortisol levels, though cortisol levels are altered in this disorder. Thus, further investigations and studies can be carried out regarding the potential role of controlling cortisol levels in the treatment of OCD.

## Cortisol and psychosis

The association between cortisol levels and psychosis has garnered significant attention due to its potential implications for understanding the pathophysiology of psychotic disorders and developing targeted treatments. Psychosis, characterized by hallucinations, delusions, and disorganized thinking, involves complex neurobiological mechanisms [149]. The HPA axis plays a crucial role in this context, with cortisol being the primary hormone released during stress. Dysregulation of the HPA axis and abnormal cortisol secretion patterns have been linked to this disease [150].

HPA axis dysfunction contributes to psychosis through several mechanisms. Hypothalamic dysfunction can lead to excessive production of CRH, which stimulates ACTH release and subsequent cortisol overproduction. Persistent hypercortisolemia may result in neuronal damage and the manifestation of psychotic symptoms. Additionally, alterations in pituitary or adrenal gland function can disrupt normal cortisol rhythms, further influencing brain function and behavior [151]. Cortisol’s effects on the brain, particularly in regions rich in GRs such as the hippocampus and PFC, are profound. Chronic cortisol exposure can lead to hippocampal atrophy, impacting memory and cognitive function, and contributing to psychotic symptomatology. Elevated cortisol levels can also impair PFC function, essential for executive function and reality testing, exacerbating symptoms like disorganized thinking and delusions [152]. Furthermore, cortisol dysregulation can trigger inflammatory processes within the brain, leading to neuroinflammation, which has been associated with the pathophysiology of psychosis [151].

Genetic and environmental interactions play a significant role in linking cortisol to psychosis. Genetic variations in HPA axis-related genes may predispose individuals to cortisol dysregulation and increase psychosis risk. Environmental stressors, such as ELS, trauma, and chronic stress, can lead to long-term alterations in HPA axis function, increasing susceptibility to psychosis [151]. Clinically, cortisol levels and HPA axis activity can serve as potential biomarkers for identifying individuals at high risk for psychosis, aiding in early diagnosis and intervention strategies. Therapeutic approaches targeting HPA axis dysregulation, such as GR antagonists or cortisol synthesis inhibitors [153], may offer new treatment avenues for psychosis. Additionally, stress management interventions, like cognitive-behavioral therapy and mindfulness-based stress reduction, could be beneficial in managing psychotic symptoms [152].

The association between cortisol dysregulation and psychosis highlights the central role of the HPA axis in the pathophysiology of psychotic disorders. Evidence indicates that elevated cortisol levels, often driven by excessive CRH and ACTH production, can contribute to structural and functional brain changes particularly in the hippocampus and prefrontal cortex [154], resulting in cognitive deficits, memory impairment, and hallmark psychotic symptoms such as disorganized thinking and delusions. Cortisol-induced neuroinflammation further supports a link between stress response and psychiatric pathology. Patients with psychotic disorders often present enhanced basal cortisol and a hyperactive HPA axis, likely to reflect chronic stress. Cortisol abnormalities correlate with the severity of symptoms and cognitive deficits linked to schizophrenia. Neuroimaging reports demonstrate that hippocampal volume reductions and prefrontal cortex dysfunction are associated with cortisol dysregulation, linking stress neurobiology with psychosis risk and progression [155].

In conclusion, the connection between cortisol and psychosis highlights the significant role of the HPA axis in the development and progression of psychotic disorders. By advancing our understanding of these mechanisms, we can improve diagnostic and therapeutic strategies, ultimately enhancing outcomes for individuals affected by psychosis (Tables 8 and 9).

**Table 8:** Studies of cortisol and psychosis relationship

Findings	Ref.
Higher diurnal cortisol levels in siblings of patients with psychosis; increased cortisol reactivity to stress linked to psychotic experiences.	[156]
A potential link between elevated cortisol levels and the risk for developing psychosis.	[149]
Potential involvement of cortisol in the pathophysiology of psychosis, but the relationship remains unclear.	[157]
Elevated diurnal cortisol and daily stressors in late childhood/early adolescence are independently associated with increased risk for developing attenuated psychotic symptoms in young adulthood.	[158]
Abnormalities in the CAR are linked to the pathogenesis of psychotic disorders; elevated stress-induced dopamine release correlates with salivary cortisol response.	[159]

Association between cortisol dysregulation and psychosis according to the previous studies

CAR: cortisol awakening response/rise

**Table 9:** Drugs targeting cortisol in treatment of psychosis

Drug class	Drug name	Mechanism of action	Effect on cortisol	Ref.
CRH antagonists	R121919	Block the action of CRH, reducing cortisol levels.	Modulating the HPA axis and lowering stress-induced cortisol responses.	[160]
GR antagonists	RU 486 [Mifepristone)	Blocks GRs, reducing cortisol effects on the brain.	Normalizing HPA axis function.	[161]
DHEA		Modulates HPA axis activity and may counteract effects of elevated cortisol.	Potential benefits in mood stabilization and reduction of psychotic symptoms.	[162]
Somatostatin analogs	Octreotide	Inhibit the release of growth hormone and reduce cortisol secretion.	Lowering cortisol levels.	[163]

Effects of R121919, mifepristone, DHEA, and octreotide on cortisol regulation for treating psychotic symptoms

CRH: corticotropin releasing hormone, HPA: hypothalamic pituitary adrenal, GR: glucocorticoid receptor, DHEA: dehydroepiandrosterone

## The cortisol axis and eating disorders

Eating disorders, notably anorexia nervosa (AN) and bulimia nervosa (BN), are among the psychiatric conditions most strongly associated with alterations in the HPA axis and cortisol dysregulation [164, 165]. Multiple studies have consistently reported elevated basal cortisol levels in patients with anorexia nervosa compared to healthy controls, a phenomenon referred to as hypercortisolemia. This elevation is thought to result from the chronic physiological and psychological stress associated with severe food restriction and malnutrition, which leads to sustained HPA axis activation. In anorexia nervosa, increased levels of CRH in the cerebrospinal fluid, elevated ACTH, and a relative resistance to dexamethasone suppression suggest impaired glucocorticoid feedback sensitivity and primary neuroendocrine abnormalities in HPA control [166, 167].

The hypercortisolemia seen in anorexia nervosa is believed to have both adaptive and maladaptive consequences. On one hand, it may represent a compensatory mechanism to maintain euglycemia in conditions of chronic undernutrition by promoting gluconeogenesis and lipolysis [168]. On the other hand, sustained high cortisol levels are associated with clinically significant outcomes such as reduced bone mineral density, cognitive deficits, insulin resistance, and increased cardiovascular risk. Notably, while cortisol levels generally decline with refeeding and weight restoration, evidence suggests that HPA axis dysregulation can persist even after apparent symptomatic recovery [169].

In bulimia nervosa and binge-eating disorder, research has revealed a more complex picture. Some studies report that these conditions can be associated with heightened cortisol reactivity to stress, while others observe blunted or hypo-reactive cortisol responses, which may persist following treatment. This blunted HPA responsiveness to stress in both AN and BN may reflect chronic allostatic overload due to repeated activation of stress systems or represent an adaptation to long-term psychopathology. Furthermore, individual differences in HPA-axis function may influence the severity, type, and prognosis of eating disorder symptoms [168, 170].

Overall, robust evidence indicates significant HPA axis dysregulation and cortisol abnormalities across the spectrum of eating disorders, particularly in AN, and to a variable extent in BN. These neuroendocrine alterations have important implications for our understanding of the pathophysiology and clinical management of eating disorders.

## Neural substrates underlying these effects, brain regions and neural circuits

Godsil et al. [171] found that patients with psychosis exhibit structural anomalies such as hippocampal volume loss and cortical thinning in the PFC. There is a marked reduction in white matter integrity within the hippocampus, PFC, and anterior cingulate cortex—a critical hub connecting limbic and cortical regions. These microstructural changes extend to the fornix, a principal white matter tract through which the hippocampus communicates with other brain areas, including the PFC. Resting-state and task-related functional MRI studies revealed aberrant functional coupling between the hippocampus and medial PFC, notably dysfunction within the brain’s default mode network, which is crucial for internally directed cognition. Neural oscillations in the theta frequency band (4–8 Hz), which normally synchronize hippocampal and prefrontal neuronal activity during working memory tasks, are disrupted in psychosis. This aberrant oscillatory synchrony is linked to cognitive deficits. Animal models simulating psychosis through maternal immune activation show decreased hippocampal-PFC theta coherence, which can be pharmacologically ameliorated by antipsychotic agents like clozapine. Dysfunction in the H-PFC pathway contributes to impaired contextual recall of fear extinction, which may underlie emotional dysregulation, delusions, and psychosis. Hippocampal overactivity may dysregulate downstream dopaminergic circuits in the nucleus accumbens, further impairing PFC function, exacerbating symptomatology. MDD is characterized by reduced hippocampal volume and metabolic abnormalities in the medial PFC, especially the subgenual anterior cingulate cortex (Cg25; part of the ventromedial PFC). This region is hyperactive during depressive episodes and normal sadness but normalizes following antidepressant treatment, placebo, or deep brain stimulation targeted to white matter beneath Cg25. Functional connectivity between the hippocampus and Cg25 is altered in depressed patients and differs between those who respond and do not respond to antidepressants, highlighting the importance of this circuit for treatment efficacy. Post-traumatic stress disorder (PTSD) patients exhibit volumetric reductions in the hippocampus, amygdala, and anterior cingulate cortex (ACC), all crucial nodes in emotional and contextual memory processing circuits. During fear extinction tasks, PTSD patients show decreased activation in the hippocampus and medial PFC but increased activity in the dorsal ACC, implicating impaired regulation within the corticolimbic fear circuitry. Alterations in amygdala and mPFC activation correspond with symptom severity, while dynamic changes in hippocampus and subgenual ACC activation relate to recovery trajectories. PTSD involves disturbed resting-state connectivity between the amygdala and ACC, as well as amygdala and hippocampus, consistent with rodent findings on the interaction of hippocampus, PFC, and amygdala in emotional regulation.

According to Battaglia et al. [172], in PTSD, cortisol levels often show an imbalance. Reduced baseline levels can coexist with dysregulated responses to stress or trauma cues. This cortisol dysregulation impairs fear extinction mechanisms, contributing to persistent and exaggerated fear responses typical of PTSD. Thus, therapeutic modulation of cortisol, for example via administration of corticosteroids like dexamethasone, can reduce cortisol levels and facilitate fear extinction, offering neuropharmacological interventions to correct abnormal fear learning.

De Alcubierre et al. [173] concluded that glucocorticoids-related illnesses are frequently associated with cognitive impairment. Memory is the most afflicted domain, with the hippocampus and PFC being the primary brain regions impacted. Although there is conflicting evidence across conditions, these individuals are at risk for cognitive impairment due to the duration of their disease, disruption of their circadian rhythm, amounts of circulating glucocorticoids, and imbalanced MR/GR activation. It is possible that glucocorticoids-dependent structural brain changes, which might continue even after long-term remission, are the cause of the failure to return to normal cognitive functioning following therapy. Cognitive deficits in patients with glucocorticoids-related disorders are challenging, often delayed, or mistaken. Prompt recognition and treatment of underlying disease may be important to avoid a long-lasting impact on glucocorticoids-sensitive areas of the brain. However, the resolution of hormonal imbalance is not always followed by complete recovery, suggesting irreversible adverse effects on the CNS, for which there are no specific treatments. Further studies are needed to find the mechanisms involved, which may eventually be targeted for treatment strategies.

Di Gregorio and Battaglia [174] found that coordinated communication between the brain and body is essential for survival, cognition, and behavior. Disruption of this brain–body balance can contribute to neurological and psychiatric diseases. Many psychiatric, neurological, and neurodegenerative disorders exhibit dysregulations in brain and body interactions. These include abnormalities in central and autonomic nervous systems, immune system, and gut microbiome, among others. Integrated assessment combining neural activity measurements, e.g., EEG, with physiological markers. e.g., heart rate variability, enhances diagnostic accuracy and understanding of disease mechanisms in neurodegeneration and psychiatric disorders. Autonomic dysfunction—characterized by increased sympathetic and decreased parasympathetic activity—is common in psychiatric disorders such as depression, anxiety, panic disorder, psychosis, and PTSD, linking autonomic imbalance to neuroinflammation and symptom progression. Furthermore, alterations in the gut microbiome contribute to maladaptive immune responses implicated in the pathogenesis of Parkinson’s disease, Alzheimer’s disease, mood disorders, and psychosis.

## Integrative conceptual models: HPA axis, neuroinflammation, gut-brain axis, and early life stress

Recent research highlights the complex interplay between the HPA axis and other biological systems - particularly neuroinflammation, the gut-brain axis, and the impact of early life stress - in the pathophysiology of psychiatric disorders

### HPA axis and neuroinflammation

The HPA axis is a pivotal regulator of the stress response, and chronic stress can lead to its persistent activation. Sustained HPA axis stimulation promotes elevated cortisol, which, under normal conditions, suppresses inflammation. However, when the HPA axis is dysregulated, glucocorticoid resistance may develop, reducing the anti-inflammatory effects of cortisol. This condition fosters a pro-inflammatory state, characterized by increased production of cytokines such as IL-6 and TNF-α, and has been observed across multiple psychiatric disorders, including depression and schizophrenia. Therefore, targeting the inflammatory processes and restoring glucocorticoid sensitivity are emerging as potential therapeutic avenues [175–177].

### The gut-brain axis

The gut-brain axis refers to the bidirectional communication network linking the gastrointestinal tract and central nervous system, mediated by the microbiota, immune system, and neuroendocrine signaling, particularly through the HPA axis. Dysbiosis, or disruption of gut microbiota, can influence the HPA axis via microbial metabolites and cytokine signaling, contributing to the neurobiology of depression, anxiety, and even schizophrenia. Conversely, chronic HPA axis activation alters gut permeability and composition. This relationship is an area of intense investigation for novel interventions, including psychobiotics and dietary strategies [178–180].

### Early life stress and allostatic load

Adverse experiences in early life, such as trauma or neglect, are major risk factors for psychiatric disorders later in life. These events can lead to long-term changes in HPA axis function, often manifesting as altered cortisol rhythms or blunted reactivity to stress, and increasing vulnerability to mental illness. The concept of allostatic load describes the cumulative physical “wear-and-tear” caused by repeated activation of the stress response system over time. Evidence suggests that the pathological effects of early life stress include not only lasting HPA axis dysregulation but also structural and functional brain changes, and heightened susceptibility to neuroinflammatory processes [176, 181].

#### Integrative perspectives

Bringing these models together underscores the intricate feedback loops among stress response (HPA axis), immune and inflammatory processes, gut microbiota, and psychosocial factors such as early adversity. These systems approach promotes a holistic understanding of psychiatric disorder etiology and fosters the development of multidisciplinary treatment strategies.

## Conclusion

The relationship between cortisol and psychiatric illnesses underscores the importance of considering hormonal factors in the diagnosis and treatment of mental disorders. Across a spectrum of psychiatric disorders—including MDD, psychosis, OCD, anxiety, ASD, ADHD, and BD—dysregulation of the HPA axis and altered cortisol levels have emerged as central but heterogeneous features in some, and not all, of psychiatric illnesses. While elevated cortisol and HPA hyperactivity are commonly and consistently linked to disorders such as MDD, psychosis, and OCD, the patterns for other diseases are far from uniform. For instance, some subgroups of individuals with ADHD and ASD show blunted or altered cortisol responses, and findings in BD remain inconsistent due to confounding variables like illness phase, treatment status, and comorbidities. Treatments that effectively target cortisol levels not only hold promise for alleviating symptoms but may also contribute to better long-term management of these complex conditions. As research continues to evolve, understanding the nuances of cortisol’s role will be crucial for developing more effective therapeutic strategies tailored to individual patient needs. Across all disorders, cortisol regulation shows substantial interindividual variability, e.g., subtype-specific patterns in ADHD, developmental sensitivity in ASD, and comorbidities in BD.

Critically, these findings emphasize the need for nuanced models that integrate cortisol dynamics with genetic, neurobiological, and environmental risk factors. Such an approach could help disentangle disorder-specific from transdiagnostic mechanisms. Therapeutically, targeting cortisol dysregulation holds promise, but clinical translation requires careful evaluation of safety, efficacy, and patient subgroup responsiveness. Precision psychiatry approaches that tailor interventions to individual cortisol profiles may ultimately improve treatment outcomes. Collectively, advancing our understanding of cortisol’s multifaceted role could bridge the gap between neuroendocrine research and personalized care in psychiatry.

Future studies should develop personalized cortisol profiling protocols, diurnal slope, CAR, and HCC, examine sex differences, age, and genetic polymorphisms, implement designs to understand how cortisol dynamics evolve over time and in response to interventions, examine HPA axis dysregulation and neuroinflammation in ASD and BD using more biomarkers such as cytokines and microglial activation, and investigate how comorbid conditions interact with HPA axis dysregulation. Table 10 offers a clearer comparative overview across disorders summarizing cortisol/HPA axis patterns. Table 11 presents the limitations of the current studies in relation to the cortisol–psychiatric disorder relationship.

**Table 10:** A comparative table summarizing cortisol/HPA axis patterns across disorders

Disorder	Associated cortisol/HPA axis pattern
ADHD	- Lower basal salivary cortisol and lower hair cortisol concentrations in children [35, 182] - Lower morning and evening cortisol levels in children and adolescents [183] - Maternal cortisol exposure in rats [184]
Autism	- Higher peak cortisol levels and prolonged duration and recovery of stress-induced cortisol elevation in children [184] - Higher basal cortisol levels, flattened diurnal cortisol rhythms, and exaggerated cortisol responses to stress and novel stimuli [185, 186]
Anxiety	- Higher salivary cortisol levels in children and adults [55, 69] - Higher cortisol levels in cattle [187] - No association in melanoma pre-op adult patients [79]
BD	- Elevated cortisol levels, disrupted diurnal rhythms, and altered HPA axis function [188]
Depression	- Increased cortisol secretion in adults, corresponding to severity [189] - Impaired cortisol negative feedback mechanism [104, 105] - Increased cortisol-induced reuptake of serotonin and reduction of tryptophan plasma levels [106, 110] - Cortisol-induced suppression of the production of brain-derived neurotrophic factor [111] - Suppressed cortisol circadian rhythms [190] - Increased CRF concentrations in the CSF, and brain CRHR-1 receptors [120, 122]
OCD	- Elevated cortisol levels corresponding to severity, bidirectional relationship [123, 139] - Increased nocturnal secretion of ACTH and cortisol [140] - Chronic low cortisol secretion [141]
Psychosis	- Elevated cortisol levels
AN	- Elevated levels of basal cortisol, corticotropin-releasing hormone and ACTH
BN	- Both heightened cortisol reactivity and blunted cortisol responses reported [191, 192]

Cortisol/HPA axis patterns across ADHD, autism, anxiety, BD, depression, OCD, psychosis, AN, and BN

HPA: hypothalamic pituitary, ADHD: attention deficit hyperactivity disorder, BD: bipolar disorder, CRF: corticotropin releasing factor, CSF: cerebrospinal fluid, CRHR-1: corticotropin-releasing hormone receptor 1 gene, OCD: obsessive compulsive disorder, ACTH: adrenocorticotropic hormone, AN: anorexia nervosa, BN: bulimia nervosa

**Table 11:** Limitations of the current studies, involving study design and sample size in relation to the cortisol–psychiatric disorder relationship

Ref.	Sample size	Study design	Measured outcomes	Limitation
[182]	129 children with ADHD, aged 6–12 years; 80 healthy controls	Cross-sectional, case–control observational study	Basal salivary cortisol levels	Cross-sectional design prevents inference of causality between ADHD and salivary cortisol
[35]	98 children with ADHD, aged 6–11 years [61boys, 37 girls); 107 healthy controls [48boys, 59 girls)	Cross sectional, case-control study	**Primary:** HCC as marker of long-term HPA activity **Secondary:** Skin conductance response as marker of sympathetic reactivity	Cross-sectional design prevents inference of causality between ADHD and cortisol
[193]	Cortisol studies: 19 studies, 916 ADHD youth, 947 healthy controls; Inflammatory biomarker studies: 4 studies, total 404 youth (257 in TNF-α subgroup)	Systematic review and meta-analysis of case-control studies	**Primary:** Basal and cumulative cortisol levels (blood/saliva) **Secondary:** Inflammatory biomarkers (TNF-α, IL-1β, IL-6, IL-10)	High heterogeneity across included studies (different methods, sample characteristics, and biomarkers assessed) limits generalizability
[37]	19 studies including participants children and adolescents with ADHD, some with comorbid CD/ODD or MDD	Systematic review	**Primary:** Cortisol stress response to acute psychosocial stress **Secondary:** Influence of comorbid CD, ODD, or MDD	Heterogeneity in study designs, stress protocols, and participant characteristics across included studies limits comparability and generalizability.
[194]	96 adults with ADHD (38 inattentive, 58 combined); 25 healthy controls	Case-control	**Primary:** Cortisol response to stress (Trier Social Stress Test) **Secondary:** Subjective stress levels (Perceived Stress Scale)	Small control group (*n* = 25) may limit statistical power to detect differences between ADHD and healthy adults
[39]	128 male children with ADHD, aged 6–14 years; 30 healthy male controls	Case-control	**Primary:** Morning plasma cortisol and ACTH levels **Secondary:** IQ via Raven’s Standard Progressive Matrices	Only male participants were included, limiting generalizability to female ADHD populations
[185]	20 children with autism (11 males, 9 females), aged 3–10 years; 28 controls (15 males, 13 females), aged3–12	Case-control	**Primary:** Serum cortisol at rest, in a novel environment, and in response to blood draw stressor **Secondary:** Autism severity (CARS), behavioral functioning (CBCL)	Small sample size limits statistical power and generalizability
[186]	32 males with ASD, aged 9–18 years	Cross-sectional (experimental stress protocol with stressor vs non-stressor tasks)	**Primary:** Cortisol concentrations during stressor and recovery phases **Secondary:** Association of cortisol recovery with social phobia symptoms and morning cortisol	All-male sample limits generalizability to females with ASD
[69]	119 children undergoing EGD with propofol, aged 5–18 years; 85 healthy controls	Observational, case-control study	**Primary:** Salivary cortisol changes (pre- and post-procedure, control) **Secondary:** Association of anxiety with cortisol, propofol dose, and procedure durations	Single-center design, limiting generalizability
[187]	31 clinical high-risk patients for psychosis (21 males; 10 females)	Cross-sectional observational study	Salivary cortisol concentration upon clinic entry	Small sample size limits statistical power and generalizability
[189]	Forty-one case-control studies included; mixed ages and illness phases.	Systematic review, meta-analysis, and meta-regression	**Primary:** Cortisol, ACTH, and CRH levels in BD vs. controls **Secondary:** Stress reactivity, genetic, molecular, and neuroimaging correlates	Heterogeneity in study methods and participant characteristics
[104]	196 heterosexual dating couples, aged 18–21 years	Cross-sectional observational study	**Primary:** Salivary cortisol response to discussion of unresolved relationship conflict **Secondary:** Association of cortisol response with depression and anxiety symptoms and diagnoses	Study focused on dating couples, limiting generalizability to other populations
[110]	32 healthy volunteers, aged (19.7–81.7 years)	Observational, cross-sectional	**Primary:** salivary cortisol over the first hour after awakening. **Secondary:** Association between morning salivary cortisol and prefrontal serotonin transporter binding	Small sample size and wide age range may limit generalizability and statistical power.
[190]	54 drug-naïve adults with Major Depression (32 males); divided into three groups: antidepressants only (*n* = 16), yoga only (*n* = 19), yoga + antidepressants (*n* = 19).	Interventional, parallel-group clinical study	**Primary:** Changes in serum BDNF levels pre- and post-intervention. **Secondary:** Changes in serum cortisol levels pre- and post-intervention; correlation between BDNF and cortisol changes.	Small sample size and short follow-up (3 months) limit generalizability and long-term conclusions.
[139]	19 studies in the systematic review; 18 studies in the meta-analysis	Systematic review and meta-analysis of case-control studies	Cortisol levels in OCD patients versus healthy controls.	Heterogeneity across studies in sampling times, measurement methods, and participant characteristics.
[140]	18 OCD patients, aged ≥18 years and 18 healthy controls	Observational, cross-sectional case-control study.	**Primary:** Basal serum cortisol levels. **Secondary:** Perceived stress (PSS-10 scores) and correlation with OCD severity	Small sample size and lack of longitudinal follow-up to assess causality.
[192]	13 female in-patients with eating disorders (Anorexia and Bulimia nervosa), aged 18–29 years; 22 female healthy controls, aged 18–46 years.	Observational, longitudinal case-control study with pre- and post-treatment assessments	**Primary:** Salivary cortisol response to Trier Social Stress Test **Secondary:** Salivary alpha-amylase, heart rate, high-frequency heart rate variability, and negative affect	Small sample size limits statistical power and generalizability
[195]	43 patients with eating disorders: 22 with anorexia nervosa and 21 with bulimia nervosa, aged 18–60 years.	Observational, cross-sectional survey	**Primary:** Resting energy expenditure via indirect calorimetry. **Secondary:** Eating disorder psychopathology (EDE-Q), general symptoms, and morning cortisol levels.	Cross-sectional design prevents causal inference between cortisol, REE, and eating psychopathology.

Limitations of the current evidence involving study design and sample size in relation to the cortisol–psychiatric disorder relationship

ADHD: attention-deficit hyperactivity disorder, HCC: hair cortisol concentration, HPA: hypothalamic pituitary adrenal, TNF-α: tumor necrosis factor-alpha, IL-1β: interleukin-1 beta, IL-6: interleukin-6, IL-10: interleukin-10, CD: conduct disorder, ODD: oppositional defiant disorder, MDD: major depressive disorder, ACTH: adrenocorticotropic hormone, IQ: intelligence quotient, CARS: childhood autism rating scale, CBCL: child behavior checklist, ASD: autism spectrum disorder, CRH: corticotropin-releasing hormone, BD: bipolar disorder, BDNF: brain-derived neurotrophic factor, OCD: obsessive–compulsive disorder, REE: resting energy expenditure, EDE-Q: eating disorder examination questionnaire

## Data Availability

No datasets were generated or analysed during the current study.

## Abbreviations

5-HT: Serotonin
ACC: Anterior cingulate cortex
ACTH: Adrenocorticotropic hormone
AN: Anorexia nervosa
ASD: Autism spectrum disorder
AVP: Arginine vasopressin
BD: Bipolar disorder
BDNF: Brain-derived neurotrophic factor
BN: Bulimia nervosa
CAR: Cortisol awakening response/rise
CRF: Corticotropin releasing factor
CRH: Corticotropin releasing hormone
CSF: Cerebrospinal fluid
DHEA: Dehydroepiandrosterone
DNA: Deoxyribonucleic acid
DST: Dexamethasone suppression test
EEG: Electroencephalogram
EGD: Esophago-gastro-duodenoscopy
ELS: Early life stress
GABA: Gamma-aminobutyric acid
GR: Glucocorticoid receptor
HCC: Hair cortisol concentrations
HPA: Hypothalamic pituitary adrenal
MDD: Major depressive disorder
MPH: Methylphenidate
MR: Mineralocorticoid receptor
MRI: Magnetic resonance imaging
NSSI: Non-suicidal self-injury
PFC: Prefrontal cortex
PTSD: Post-traumatic stress disorder
SCL: Salivary cortisol levels
SHR: Spontaneously hypertensive rats
SNPs: Single-nucleotide polymorphisms
SSRIs: Selective serotonin reuptake inhibitors
